# British Health Stations.—IV

**Published:** 1904-07-30

**Authors:** 


### BRITISH HEALTH STATIONS.—IV.

(By Our Special Medical Commissioner.)

### THE CORNISH PENINSULA.

The delectable Duchy, as the county of Cornwall has been called, has long been known to the artist and archæologist as a land rich in delights for the mind, and to the enterprising pedestrian and far-wandering holiday-maker, the characteristics of the district making for physical invigoration have long been known. But it is only comparatively recently that any well-defined effort has been made to bring this natural sanatorium of the West to the knowledge and reach of those seeking health. At last, thanks to railway enterprise, the development of means for locomotion by motors and coaches, the establishment of modern hotels, and a considerable awakening of local enterprise in other respects, Cornwall has been rendered a particularly suitable resort for the invalid, convalescent, debilitated, and overworked. It is our purpose to briefly indicate the more important physical climatic and general characters of the county, which should make it take a foremost place among our British health stations.[1]

Access to Cornwall has now been rendered rapid and comfortable, so that not even the most delicate may be deterred by the length or discomforts of the journey.[2] The Great Western Railway runs trains composed of corridor dining cars and carriages fitted with all the requisites of an invalid, from Paddington to Penzance, and now a journey without any stop is made from London to Plymouth. Even on the branch lines, the comfort and convenience of the public have been considered, and various important districts peculiarly suited to the necessities of certain cases have been opened up by a regular system of comfortable motor cars. The London and South-Western Railway supplies the north-eastern portion of the county, and through carriages from Waterloo, *via* Exeter, run to Padstow and Bude, and by arrangement with coaches allow of comfortable access to Newquay, Tintagel, and Boscastle. Doubtless this company will follow the example of the Great Western and open up other districts by means of convenient motor cars.

Cornwall, viewed from the health aspect, may be looked upon as consisting of three parts; a broad backbone of more or less bare, almost uninhabited, windswept tableland, with an elevation varying from 300 to 700 feet; a northern slope limited by a wild, rocky, picturesque coast-line, and open to the north-west winds from the Atlantic; and a southern slope, with much indented coastline and bold rugged rocks composed of granite in the south-west portion, and of serpentine in the Lizard district.[3]

The climatological conditions are such as peculiarly fit Cornwall as a desirable health station for a wide variety of cases. Its temperature is characterised by equability. The annual mean temperature of eight of its more important centres is 50·1 degrees, while for the months of January, February, and March, the mean temperature is 42·6 degrees. There is also but slight daily variation. It is interesting here to note that, during the autumn and winter, the temperature of the sea round the Cornish coast is distinctly warmer than that of the atmosphere. The rainfall in Cornwall is high, its eight mainland stations giving an average of 38·9 inches for the year; but the soil allows of much dryness, and such mists as prevail are generally from the sea. The degree of humidity is such as to afford a sedative action on many complaints. The amount of sunshine that prevails seems to be greater than at most health resorts. The southern and south-western districts, in particular, enjoy much sunshine throughout the year. The prevailing winds are from the south, west, and north, while the easterly winds are of less frequency and usually considerably modified in force and character. Many of the health stations, to which we hope subsequently to refer, are so placed as to afford protection from winds.

The geological features of the county are of much interest, and account to a great extent for many of its desirable health-giving characteristics. Numerous springs afford abundant supply of clear, pure, and peculiarly soft water. Calcareous matter is rare, and so-called calculous disorders are infrequent.

In studying returns as to the pathology of Cornwall, the influence of occupation must not be forgotten, for a large proportion of the natives are miners or fishermen. Generally speaking, but little actual illness prevails. Zymotic disease is comparatively rare. Rheumatism is somewhat common, and diabetes is said to occur in a greater ratio than in all England taken together. Renal disease is below the general average. Death from diseases of the respiratory tract occur in smaller proportion than in England as a whole. The deaths from pulmonary tuberculosis are low on the north coast, but in certain of the mining districts phthisis is very prevalent.[4]

No sanatorium for the tuberculous has at present been established in the county, although certain districts are eminently suited for open-air treatment. It is generally considered, although perhaps on not altogether adequate grounds, that rheumatic cases do not do well. Skin affections, especially eczema, should not be sent as a rule to the coast towns. Chronic cardiac cases, when not of rheumatic origin, should do well in some of the sheltered resorts. It seems likely that certain places on the north coast should offer many

[1] See also "The Climate of Cornwall." By W. Howship Dickinson, M.D., F.R.C.P., in the "Climates and Baths of Great Britain." Vol. i. London: 1895.

[2] "The Cornish Riviera." London: Great Western Railway Offices, Paddington.

[3] Consult Baddeley and Ward's excellent Guides to Devon and Cornwall, with admirable maps; North and South published in separate volumes. London: Dulau and Co. A useful and up-to-date Guide to North Cornwall is published by C. Arthur Pearson, Ltd. Messrs. Ward, Lock and Co. issue convenient guide-books to various Cornish resorts. Messrs. Adam and Charles Black, and Mr. John Murray also publish Guides to Cornwall. See also "Highways and Byways in Devon and Cornwall." By Arthur H. Norway. London: Macmillan and Co. 1898; and certain admirable handbooks published by The Homeland Association, 24 Bride Lane, Fleet Street, London, E.C. Much interesting local information likely to be of service to the thoughtful visitor will be found in the volumes of the now defunct "Cornish Magazine," edited by A. T. Quiller-Couch ("Q."). Truro: Joseph Pollard.

[4] See "Annual Report, Vital Statistics, and Meteorological Summary for 1903." Cornwall County Council. Truro: 1904.

advantages to anæmic and so-called scrofulous children, and for those, and the subjects of many forms of surgical tuberculosis, Newquay and adjacent parts experience will probably show to be as beneficial as Margate or other well-known resorts.

With the awakening of the Duchy to its almost limitless possibilities as a desirable health resort for widely differing classes of patients, it may be hoped that local enterprise will be forthcoming to provide all those adventitious elements which make for the comfort and general well-being of the health-seeker and holiday-maker.[5]

